# Dietary advice and oral nutritional supplements do not increase survival in older malnourished adults: a multicentre randomised controlled trial

**DOI:** 10.1080/03009734.2020.1751752

**Published:** 2020-05-02

**Authors:** Lisa Söderström, Andreas Rosenblad, Leif Bergkvist, Hanna Frid, Eva Thors Adolfsson

**Affiliations:** a Centre for Clinical Research, Region Västmanland, Uppsala University, Västerås, Sweden;; b Department of Food Studies, Nutrition and Dietetics, Uppsala University, Uppsala, Sweden;; c Department of Statistics, Stockholm University, Stockholm, Sweden;; d Department of Medical Sciences, Clinical Diabetology and Metabolism, Uppsala University, Uppsala, Sweden;; e Department of Child and Adolescence Psychiatry, Västmanland Hospital, Västerås, Sweden

**Keywords:** Dietary advice, malnutrition, older adults, oral nutritional supplementation, randomised controlled trial, survival analysis

## Abstract

**Objectives:** The study aimed to investigate the effect on survival after 6 months of treatment involving individual dietary advice and oral nutritional supplements in older malnourished adults after discharge from hospital.

**Methods:** This multicentre randomised controlled trial included 671 patients aged 65 years who were malnourished or at risk of malnutrition when admitted to hospital between 2010 and 2014, and followed up after 8.2 years (median 4.1 years). Patients were randomised to receive dietary advice or oral nutritional supplements, separate or in combination, or routine care. The intervention started at discharge from the hospital and continued for 6 months, with survival being the main outcome measure.

**Results:** During the follow-up period 398 (59.3%) participants died. At follow-up, the survival rates were 36.9% for dietary advice, 42.4% for oral nutritional supplements, 40.2% for dietary advice combined with oral nutritional supplements, and 43.3% for the control group (log-rank test *p* = 0.762). After stratifying the participants according to nutritional status, survival still did not differ significantly between the treatment arms (log-rank test *p* = 0.480 and *p* = 0.298 for the 506 participants at risk of malnutrition and the 165 malnourished participants, respectively).

**Conclusions:** Oral nutritional supplements with or without dietary advice, or dietary advice alone, do not improve the survival of malnourished older adults. These results do not support the routine use of supplements in older malnourished adults, provided that survival is the aim of the treatment.

**Trial registration:** ClinicalTrials.gov with ID: NCT01057914

## Introduction

Malnutrition is still a common problem in older adults in any setting (1), and the condition is associated with many negative health outcomes (2–5), including mortality (6–8). Nutritional treatment strategies aiming at increasing survival among older adults is highly relevant, since the life expectancy at 65 years is around 20 years in Sweden and many other countries. However, the effectiveness of nutritional interventions is still uncertain (9). Evidence suggests that dietary advice and oral nutritional supplements may help maintain body weight (10) or cause a modest weight gain (9,11,12), and improve body composition (11–13) and grip strength (11,12), but the effect on survival is unclear (9,11,12,14).

Mortality is considered a critical outcome to analyse when evaluating nutritional interventions aiming at preventing or treating malnutrition in older adults (15). All-cause mortality is preferred over cause-specific mortality as an outcome measure, since it avoids such problems as misclassification bias related to the true cause of death (16). Moreover, it balances out the harmful and beneficial impacts an exposure may have on health, thus giving the net effect of an exposure on mortality.

The results from a Cochrane review (14) including data from 42 randomised controlled trials (RCTs) in older adults (aged 65 years) with different nutritional statuses reported no reduced mortality in groups that received oral nutritional supplements compared with a control group. Subgroup analyses indicated an effect in older adults who were already malnourished (21% reduction in mortality rate) and older adults who received oral nutritional supplements of 400 kcal a day (11% reduction in mortality rate). However, no single trial has had sufficient statistical power or length of follow-up to investigate mortality as a primary outcome. Additional data from large-scale multicentre trials are required to strengthen the evidence base.

The present multicentre RCT included older adults with malnutrition, or at risk of malnutrition, with the aim of investigating the effect on survival after 6 months of intervention consisting of individual dietary advice, oral nutritional supplements, separately or in combination, or routine care. The hypothesis was that the survival differed between the intervention groups and the control group.

## Materials and methods

### Study design and setting

This was a multicentre RCT involving three intervention groups that received individual dietary advice, oral nutritional supplements, or a combination thereof. A fourth group served as the control group. The patients were recruited to the study when admitted to an internal medicine, surgical, or orthopaedic ward, for miscellaneous reasons, at five small-to-large-scale hospitals in central Sweden between February 2010 and December 2014. The trial was conducted by registered dietitians. The dietitians engaged in the study were employed by the participating hospitals. Some were recruited to work with the study directly after their dietetic studies, while others had been working for several years. Before they started to recruit patients, the dietitians received 2 days of instruction about the study protocol, including how to use the Mini Nutritional Assessment (MNA) instrument, by the project leaders. Thereafter, the dietitians had weekly telephone conferences where they could discuss any uncertainties or other issues that had arisen. Twice a year during the study period the dietitians met in person and practised the implementation of the study protocol, to decrease inter-rater variability.

### Participants

**Figure 1.**
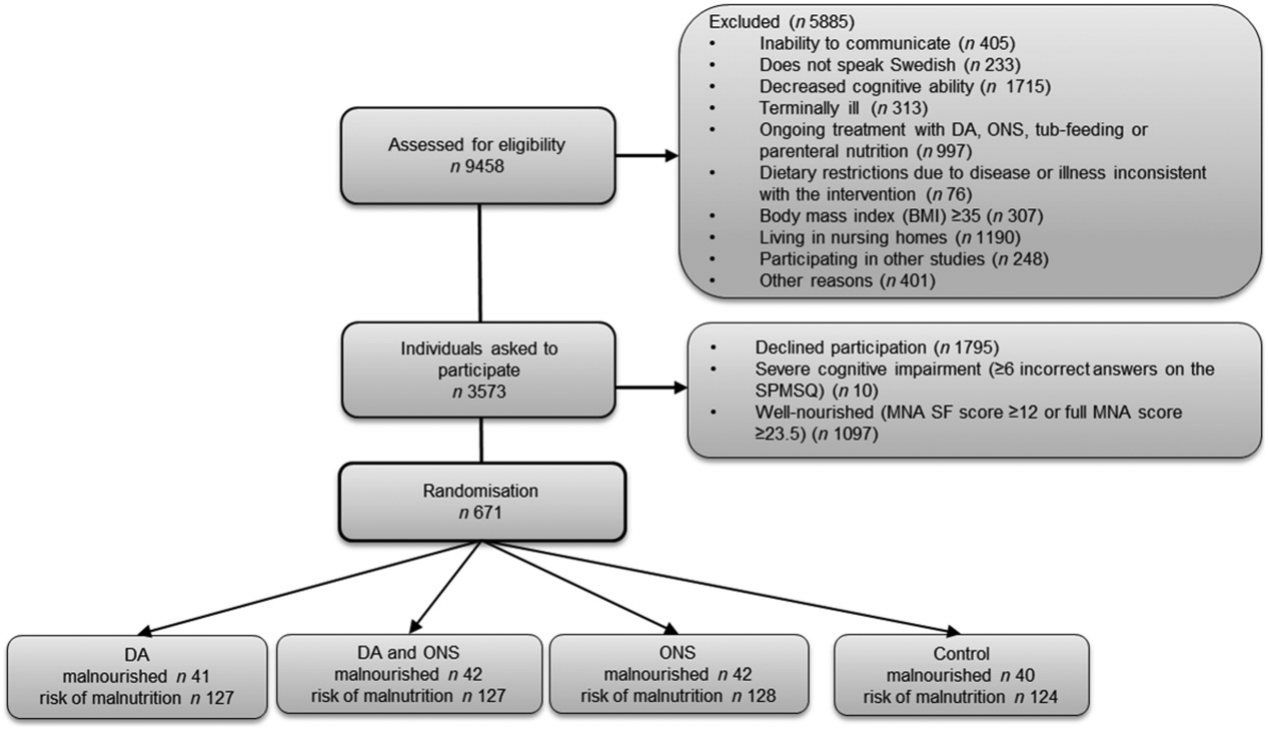
Flow chart describing the participant recruitment and randomisation process in five hospitals in central Sweden. DA: dietary advice; MNA: Mini Nutritional Assessment; MNA SF: MNA Short Form; ONS: oral nutritional supplements; SPMSQ: Short Portable Mental Status Questionnaire.

Patients aged 65 years were included (Figure 1). The patients were informed both verbally and in writing about the study by the dietitians and were then asked to participate. They were screened with the MNA instrument (17–19) and the Short Portable Mental Status Questionnaire (SPMSQ) (20–22). The MNA has been validated in previous studies and been shown to predict mortality (8,23,24). The primary inclusion criterion was malnutrition or risk of malnutrition as indicated by a full 18-item MNA score of 23.5. Participants also had to have five incorrect answers on the SPMSQ, indicating no or at most moderate cognitive impairment. The exclusion criteria were: inability to communicate, does not speak Swedish, decreased cognitive ability, having a body mass index (BMI) ≥35 kg/m^2^, receiving a dietary intervention, living in a nursing home, or having an expected survival of <1 year. Since the expected survival was hard to estimate, in practice only those who were given palliative support were excluded according to the last-mentioned criterion.

### Randomisation procedure

A computerised block randomisation procedure with random block sizes varying between 8 and 32, stratified on nutritional status (malnourished or at risk of malnutrition), was performed by the responsible statistician (A.R.). The randomisation sequences were placed in sequentially numbered and sealed opaque envelopes by the project leader (H.F.), and the envelopes were distributed to and kept at the participating hospitals. The dietitian asked a research assistant who was not otherwise involved in this study to open the envelope and inform the dietitian which intervention group the participant was allocated to. The intervention was not blinded since this was not possible for practical reasons.

### Intervention and control groups

Information about the intervention was given during the hospitalisation, and the participants were told to start the intervention at discharge (intervention groups) or continue their usual habits (control group).

#### Dietary advice group

Patients randomised to receive dietary advice were counselled by a registered dietitian before they were discharged from hospital, and no further appointments were given once they had returned home. The patients were asked to describe their dietary habits and intake, and to discuss possible improvements with the dietitian to optimise the diet according to national dietary recommendations (25). The advice was semi-standardized and was based on the answers given in the MNA. Table 1 displays the dietary advice given to participants in the intervention groups that received dietary advice or dietary advice + oral nutritional supplements (*n* = 337).

**Table 1.** Dietary advice given to participants based on the answers from the mini nutritional assessment instrument.

	Advice aiming at improving …				Other	Advice regarding …			
MNA question with low scores	Energy density^a^	Nutrient density^b^	Texture of food	Number of meals per day^c^	Advice to relatives	How to complete meals	Protein-dense foods	Increased vegetable or fruit intake	Increased fluid intake
Declined food intake	✓	✓	✓	✓					
Recent weight loss	✓		✓	✓					
Mobility					✓				
Neuropsychological problems					✓				
Body mass index	✓			✓					
Living independently					✓				
Number of full meals						✓			
Protein intake							✓		
Vegetable intake								✓	
Fluid intake									✓
Mode of feeding					✓				
Mid arm circumference	✓		✓						
Calf circumference	✓	✓	✓						

^a^Energy density: dietary advice regarding energy supplementation with energy-dense food, e.g., butter, margarine, full-fat dairy products (milk/yoghurt/cream), oil, and sugar. Beverages should be energy-dense.

^b^Nutrient density: improved content of protein, vitamins, and minerals in meals.

^c^Number of meals per day: the energy and nutrient content should be distributed into 3 main meals and 1–3 in-between meals. The overnight fast should not exceed 11 h.

The semi-standardized approach was used to minimise interpersonal differences between the dietitians. It was developed by the project-leading dietitians and the dietitians working with the recruitment in accordance with national guidelines. The patient received a written copy of the advice. To increase the consensus between the dietitians’ dietary advice, a telephone conference was held each week during the recruitment period.

#### Oral nutritional supplements group

All patients randomised to oral nutritional supplements were asked to drink 1–2 bottles per day, depending on the energy content of the supplement, to provide 400 kcal/day and 12–20 g protein. The participants were allowed to choose between different flavours and brands to increase their compliance. Protein-dense supplements with a complete vitamin and mineral content were offered first. Only if these were not tolerated were the participants offered supplements with lower protein content. The supplements had a volume of 125–200 ml/bottle, energy density 1.25–2.4 kcal/mL, and protein content 4–9.4 g/100 ml. The oral nutritional supplements were paid for by grants unrelated to the manufacturers.

#### Combined group

The patients received dietary advice as described above for the dietary advice group. In addition, they were encouraged to drink the oral nutritional supplements in the same way as the oral nutritional supplements group.

#### Control group

The patients were informed about the screening result and that the dietitian in the study would not give any further instructions about their nutrition. However, they were free to contact a health-care professional if they were concerned about their nutritional status.

The control group was contacted by a dietitian by telephone at 1, 3, and 6 months after discharge to answer questions about health-care consumption and side effects to be able to compare possible side effects with the intervention groups.

### Follow-up

All four groups were asked questions according to a question guide, specifically developed for the present study, with both closed and open-ended questions about their visits to a general practitioner, district nurse, or dietitian, whether the patient had home care, and, if so, to what extent. The participants also answered questions about side effects such as nausea, vomiting, diarrhoea, constipation, and other problems in the gastrointestinal tract. The three intervention groups were contacted by the dietitian by telephone at 1, 3, and 6 months after discharge to check their compliance with the treatment. To assess the compliance to the oral nutritional supplements, the dietitian asked the participants if they had consumed the prescribed number of supplements at 1, 3, and 6 months. Following the same question guide, participants had the opportunity to ask questions, receive new dietary advice, or change the flavour or type of oral nutritional supplementation, if needed. Controls were contacted by the dietitian at the same intervals as the intervention groups to minimise the risk of bias caused by increased attention given to the intervention groups.

### Outcome

Survival of the intervention groups and the control group was followed up through the Swedish population register until 16 April 2018, i.e., between 3.4 years and 8.2 years after starting the nutritional treatment.

### Ethical considerations

The study was approved by the Uppsala Ethical Review Board (approval number: 2009/203). Before the patients entered the study, all provided written informed consent. All patients received at least routine treatment at the hospital. However, some participants received information about their risk of malnutrition, which gave them the opportunity to consider whether they needed or wanted to take further actions, such as consulting a dietitian. Participation in the RCT was registered in each patient’s medical record along with information on the purpose of the study and to which intervention group the patient was randomised. The trial is registered at ClinicalTrials.gov with ID: NCT01057914.

### Statistical analyses

Descriptive statistics are given as frequencies and percentages, *n* (%), for categorical data and as means and standard deviations (SDs) for discrete and continuous data. An intention-to-treat (ITT) approach was used for the analyses. Six participants were misclassified in terms of their nutritional status group; four were categorised as malnourished but had only a risk of malnutrition, and two were classified as being at risk of malnutrition but were malnourished. These people were analysed according to their allocated group.

Differences in survival between the four randomisation groups were tested using log-rank tests, stratified according to nutritional status group, and illustrated using Kaplan–Meier plots. To examine the effect of the dietary advice and oral nutritional supplements on all-cause mortality, Cox proportional hazards (PH) regression models stratified according to nutritional status group were used with time to death as outcome and dietary advice and oral nutritional supplements as predictors. This allowed the whole sample to be included since the combined (dietary advice + oral nutritional supplements) group contributed to the effects of both the dietary advice and the oral nutritional supplements predictors, thus giving a better power. The results are presented as hazard ratios (HRs) with accompanying 95% confidence intervals (CIs). As potential confounders, the following baseline variables were considered: age (years), sex (men/women), BMI, smoking (never/former/current), living alone (yes/no), length of overnight fast (hours), cooks independently (always/sometimes/never), receiving home care service (yes/no), Charlson Comorbidity Index, and number of medications.

Length of overnight fast was defined as the time between the last eating episode in the evening and the first eating episode the morning after. These variables were chosen because of their potential association with mortality (7,26). The proportional hazards (PH) assumption of the Cox regression model was tested separately for each included explanatory variable using the Grambsch–Therneau test (27). Variables that failed this test were included as piecewise variables with change point at 2 years (731 days) of follow-up, in which case they did not fail the test any more.

The statistical analyses were performed using IBM SPSS Statistics 24 and R 3.5.0, with *p* values <0.05 considered statistically significant.

### Sample size calculation

The sample size calculation was based on two studies with similar design, in which the mortality was approximately 20% in untreated patients and 10% in patients who had received nutritional treatment (10,28). To detect a 10-percentage point difference in mortality while obtaining a power (1–) of 80% at a two-sided significance level of 0.05 with a *Z* test for difference in percentages, we calculated that each group should include at least 199 patients. With mortality as end-point, the drop-out is negligible since mortality is followed up in registers. Accordingly, we calculated that 800 patients were needed for the study. A tentative interim analysis was performed after 560 participants had been included. This was not planned at the start of the study but decided upon when recruitment was slow and we needed to know if it was meaningful to continue. The analysis showed that there were no significant differences in mortality between the intervention and control groups. To obtain a statistically significant difference, an extreme difference in mortality in the remaining group of 240 patients would have been needed. Since this seemed unlikely, we decided to terminate the study early. For logistical and personnel reasons, the termination was set to December 2014. At that time, a total of 671 individuals had been included in the study, which formed the study population of the present study.

## Results

### Participant characteristics

In total, 671 patients were included in the present study. The median age was 79.0 (mean 78.7, SD 7.7) years with a range of 65–96 years, and 61% (*n* = 410) were women. The mean BMI was 24 (SD 4) kg/m^2^, and 10% (*n* = 67) were current smokers. Most of them (81%, *n* = 529) had normal cognitive functioning according to the SPMSQ, and only 16% (*n* = 107) and 3% (*n* = 20) had mild or moderate cognitive impairment, respectively. Table 2 shows the baseline characteristics of the participants grouped according to the four treatment arms. The groups differed slightly in terms of age and the variable ‘receiving home care service’. Of the excluded patients 56% were women, while the mean age was 80.6 years and the mean BMI 25.6 kg/m^2^.

**Table 2.** Characteristics of the 671 participants at baseline.

	DA	ONS	DA and ONS	Control
	(*n* = 168)	(*n* = 170)	(*n* = 169)	(*n* = 164)
Variables				
Malnourished, %	24.4	24.7	24.9	24.4
Age, y – mean (SD)	79.9 (7.9)	77.6 (7.5)	79.0 (7.6)	78.2 (7.7)
Women, %	58.3	65.9	58.6	61.6
Body mass index, kg/m^2^ – mean (SD)^a^	23.7 (3.8)	24.2 (4.2)	23.8 (4.1)	23.7 (4.2)
Smoking, %				
Never smoker	47.3	44.1	42.6	43.9
Former smoker	43.7	47.1	45.0	46.3
Current smoker	9.0	8.8	12.4	9.8
Living alone, %	55.1	44.7	49.1	49.4
Length of overnight fast, h – mean (SD)	12.4 (1.9)	12.5 (1.9)	12.3 (1.8)	12.3 (2.0)
Cooks independently, %				
Always	42.5	39.4	43.8	45.7
Sometimes	44.3	46.5	45.6	40.2
Never	13.2	14.1	10.7	14.0
Receiving home care service, %	31.3	22.6	27.2	17.8
Charlson Comorbidity Index – mean (SD)	1.2 (1.4)	1.4 (1.8)	1.3 (1.6)	1.5 (1.8)
Number of medications – mean (SD)	6.9 (3.7)	6.9 (3.7)	6.9 (3.8)	6.7 (3.7)

All characteristics except survival rate are as per baseline in 2010–2014.

^a^Two participants had a BMI ≥35 kg/m^2^.

DA: dietary advice; ONS: oral nutritional supplements; SD: standard deviation.

### Overall survival

**Figure 2.**
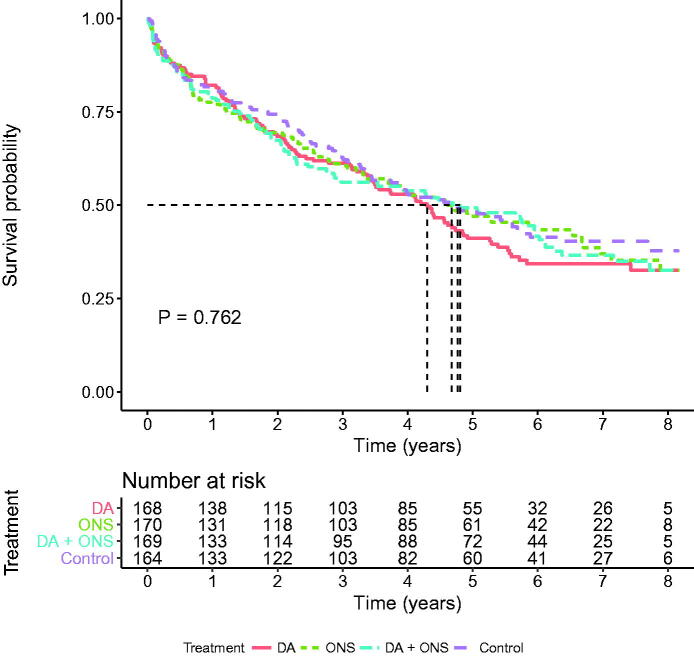
Kaplan–Meier survival curves for participants in the four intervention groups (*n* = 671). Log-rank test for any difference between groups (*p* = 0.762). DA: dietary advice; ONS: oral nutritional supplements.

**Figure 3.**
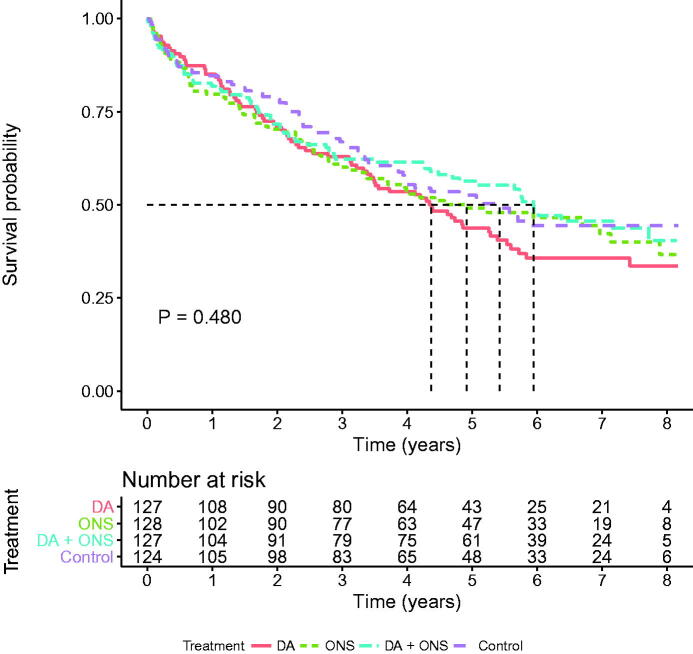
Kaplan–Meier survival curves for participants at risk of malnutrition in the four intervention groups (*n* = 506). Log-rank test for any difference between groups (*p* = 0.480). DA: dietary advice; ONS: oral nutritional supplements.

**Figure 4.**
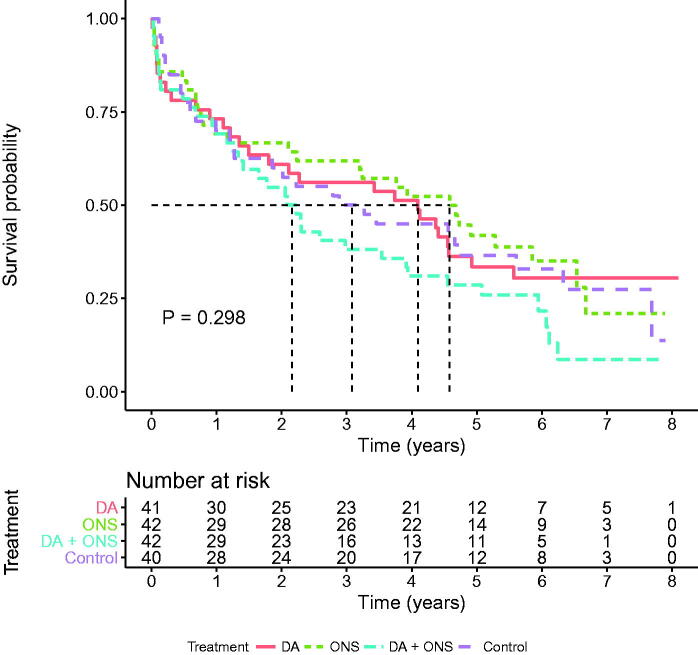
Kaplan–Meier survival curves for malnourished participants in the four intervention groups (*n* = 165). Log-rank test for any difference between groups (*p* = 0.298). DA: dietary advice; ONS: oral nutritional supplements.

The median follow-up time was 4.1 years (3.4–8.2) generating 2588 person-years. At 6 months, i.e., when the intervention was completed, a total of 94 (14%) patients had died, an equal proportion in each arm. At the end of follow-up 273 (40.7%) were still alive. The survival rate was 36.9% in the dietary advice group, 42.4% in the supplement-only group, 40.2% in the combined group, and 43.3% in the control group. The differences, however, did not attain statistical significance (log-rank test *p* = 0.762). Kaplan–Meier curves illustrate survival in the whole group (Figure 2), the malnourished group (Figure 3), and the group at risk of malnutrition (Figure 4). There were, however, no statistically significant differences. Unadjusted and adjusted hazard ratios for the respective groups are displayed in Table 3.

**Table 3.** All-cause mortality for the three interventions and the control group.

Nutritional status	Intervention	Unadjusted HR (95% CI)^a^	*p* Value	Adjusted HR (95% CI)^b^	*p* Value
All participants	DA	1.16 (0.88–1.53)	0.296	1.21 (0.83–1.75)	0.317
	ONS	1.05 (0.79–1.39)	0.749	1.08 (0.73–1.60)	0.691
	DA + ONS	1.08 (0.81–1.43)	0.603	1.08 (0.74–1.56)	0.693
	Control	1.00^c^		1.00^c^	
At risk of malnutrition	DA	1.24 (0.89–1.72)	0.201	1.20 (0.83–1.74)	0.342
	ONS	1.11 (0.79–1.55)	0.553	1.08 (0.73–1.60)	0.688
	DA + ONS	0.98 (0.70–1.39)	0.929	1.08 (0.74–1.56)	0.691
	Control	1.00^c^		1.00^c^	
Malnourished	DA	0.97 (0.58–1.64)	0.918	1.01 (0.53–1.92)	0.984
	ONS	0.90 (0.53–1.52)	0.686	1.22 (0.64–2.33)	0.538
	DA + ONS	1.39 (0.85–2.29)	0.193	1.03 (0.53–2.01)	0.931
	Control	1.00^c^		1.00^c^	

Results of separate Cox regression analyses for the participants at risk of malnutrition and the malnourished participants, respectively.

^a^Results based on 506 (100.0%) participants at risk of malnutrition and 165 (100.0%) malnourished participants.

^b^Adjusted for age, sex, body mass index, smoking, living alone, length of overnight fast, cooks independently, receiving home care service, Charlson Comorbidity Index, and number of medications. Age is included as piecewise with change point at 731 days of follow-up for both nutritional status groups, as are smoking, cooks independently, and number of medications for the malnourished group. For the total group, age is included as piecewise with change points at 731 and 1461 days of follow-up. The results are based on 423 (83.6%) participants at risk of malnutrition and 125 (75.8%) malnourished participants with complete values for all variables.

^c^Reference category.

DA: dietary advice; HR: hazard ratio; ONS: oral nutritional supplements.

### Compliance

The three intervention groups were contacted by the dietitian by telephone at 1, 3, and 6 months after discharge to check their compliance with the treatment. The self-reported compliance rates for the two groups who received oral nutritional supplements (including both malnourished patients and patients at risk of malnutrition) were 79% (*n* = 215) at 1 month, 79% (*n* = 194) at 3 months, and 74% (*n* = 157) at 6 months. In total, 70% (*n* = 104) in the group at risk of malnutrition and 63% (*n* = 34) in the malnourished group reported taking the oral nutritional supplements according to the prescribed amount at each of the three follow-up times. The highest compliance rate (74%) to the prescribed supplements was observed among malnourished individuals in the intervention group who received both dietary advice and oral nutritional supplements (*n* = 20).

There were no significant differences in side effects such as nausea, vomiting, diarrhoea, constipation, and other problems in the gastrointestinal tract between the intervention groups and the control group, and there were no significant associations between compliance and reported side-effects at telephone interview 1, 3, and 6 months after discharge.

## Discussion

The findings from this multicentre RCT imply that use of oral nutritional supplements and dietary advice does not improve survival in older adults with malnutrition or at risk of malnutrition. This confirms and adds to previous research pointing in the same direction (9,11,12,14,29).

No single trial has had sufficient statistical power or length of follow-up to investigate mortality as a primary outcome (14). Two Cochrane reviews that examined the effect of oral nutritional supplements on mortality reported conflicting results. In a 2005 review that included data from 32 trials, the mortality was reduced by 26% in the supplemented group compared with the control groups (30). Almost half of the trials did not report an ITT analysis. The authors concluded that there may be beneficial effects of supplementation on mortality, although doubt remains due to the poor quality of most of the included trials. In fact, excluding the largest study (31), which also had the lowest-quality rating, made the overall results non-significant (32). In an updated review from 2009 including data from 42 RCTs in older adults with varying nutritional statuses, no reduced mortality was detected in the group that received oral nutritional supplements compared with a control group (14). Most of these trials (60%) did not report an ITT analysis (14,30).

The conflicting results regarding mortality may be explained partly by inclusion of the multicentre FOOD trial in the 2009 meta-analysis. The FOOD trial provided oral nutritional supplements or the ordinary hospital diet to stroke patients who were mainly well-nourished. No improved survival was detected at the 6-month follow-up (33). Since the FOOD trial included 4023 patients and thus constituted 50% of the study population in the 2009 meta-analysis, it may have diluted any potential treatment effects (14). Subgroup analyses in the 2009 Cochrane review indicated that a reduction in mortality applied only to subgroups of older adults who were already malnourished and older adults who received 400 kcal/day of oral nutritional supplements (14). A systematic review from 2019 including one study with 100 elderly subjects found no effect on mortality of oral nutritional supplements, or dietary advice in combination with oral nutritional supplements in older people (29).

### Strengths and limitations of this study

This study had several methodological strengths. The patients were recruited from a wide range of hospitals in central Sweden, came from the general population, and had a variety of geriatric conditions, all of which help to increase the generalizability. However, the study population in the present study is less frail than the average population of older adults admitted to hospital, as the study only included patients who were living at home (not in a nursing home), had no cognitive impairment, and were not terminally ill.

Randomisation was performed centrally, was secure, and involved concealment of the allocations. The follow-up of deaths was almost 100% complete (one participant emigrated). This is so far one of the largest multicentre RCTs examining the effect of oral nutritional supplements and dietary advice on mortality. In a Cochrane review including data about mortality from 42 RCTs of older adults receiving nutritional treatment, the study populations in most studies numbered fewer than 100 participants (14). Moreover, most previous studies were conducted during a hospital stay or in nursing homes (10,11,14). In the Cochrane review, 85% of the patients were admitted to hospital or lived in a nursing home during the treatment. However, today most older adults in Sweden live at home. Therefore, another strength was that the effect of nutritional treatment was studied in older people living at home.

Moreover, in the aforementioned Cochrane review the duration of the intervention varied from 2 weeks to 18 months, and the follow-up time was in general the same as the duration of the treatment (14). In the present study, the duration of nutritional treatment was 6 months, with follow-up of survival for up to 8 years. Registered dietitians at each centre performed the nutritional screening and the interventions. Compliance with oral nutritional supplements was followed up by telephone and recorded by the dietitians at three time points during the treatment period. The compliance with dietary supplements in the present study was high and is consistent with an overall mean compliance of 78% (37–100%) reported in previous studies (34). In our study, malnourished individuals in the intervention group who received both dietary advice and oral nutritional supplements had the highest compliance with prescribed supplements (74%).

The present study could be criticised for not obtaining information on nutritional outcomes (e.g., weight change) during treatment and follow-up. However, given the absence of evidence for effects on our primary outcome (survival), the relevance of any effect on such surrogate outcomes is questionable. To use weight change as a measure of compliance to supplements or as an outcome is problematic, since it is only possible to measure weight on those participants who have survived until the day of follow-up, thus introducing severe survivorship bias. The relevance of mortality as a primary outcome is further stressed in a systematic review by a panel of geriatricians and experts in nutrition. Besides mortality, morbidity, and functional status, nutritional status and quality of life were considered a critical outcome for research in nutrition interventions for the prevention and treatment of malnutrition in older people (15). A further limitation was that the adherence to oral nutritional supplements and dietary advice was only followed up during the 6-month treatment period, which could weaken the reliability of the results during the long-term follow-up.

A further limitation of our study was that we did not reach our primary goal for inclusion. Because the recruitment rate was slower than expected, a tentative interim analysis was performed after about 70% of the planned number of participants had been included. The interim analysis showed no difference in survival between the exposure groups, despite a higher than expected mortality in all groups. An extreme difference in outcome among the remaining 30% of planned inclusions would have been needed to change the overall results. This was considered unlikely, and we decided to end recruitment at the end of 2014. However, the results are still of interest, pointing to the large number-needed-to-treat for the interventions considered in the present study.

A possible explanation of why survival did not differ significantly between the intervention groups and the control group could be that the standard care of malnourished patients at the hospitals fully met the nutritional needs of the patients. In the dietary advice group (*n* = 168) and control group (*n* = 164), a total of 38 individuals had visited a dietitian other than those in the current study. This was significantly more visits to the dietitians compared to the two groups that received oral nutritional supplements.

Another concern may be that although the MNA has been shown to predict a high risk of early death among individuals that have been classified as malnourished or at risk of malnutrition according to the MNA criteria, no one has shown that nutritional support to these groups may alter the cause of these conditions.

## Conclusions

We could not confirm the anticipated benefit on survival from oral nutritional supplements or dietary advice. Contrary to our hypothesis, oral nutritional supplements with or without dietary advice, or dietary advice alone, did not improve the survival in older adults who were malnourished or at risk of malnutrition. These results do not support the unselective, routine use of oral supplementation in older malnourished adults in the general population, if survival is the aim of the treatment. However, this does not mean that oral nutritional supplements should not be used at all, since the supplements may have other beneficial effects such as increased quality of life or increased performance in activities of daily living. However, the indication for providing the oral nutritional supplements must be clearly understood, both by the patient and the health-care professionals. It should also be noted that several factors, such as environmental factors and social isolation, influence nutritional intake. These factors might thus be important to include in future studies.
